# Incidental Finding of an Aneurysm of the Sinus of Valsalva in a Young Woman: A Management Dilemma

**DOI:** 10.7759/cureus.102199

**Published:** 2026-01-24

**Authors:** Marc T Zughaib, Keyur Patel, Andrew D Assaf, Marcel E Zughaib

**Affiliations:** 1 Cardiology, Henry Ford Providence Hospital, Southfield, USA; 2 Internal Medicine, Henry Ford Providence Hospital, Southfield, USA

**Keywords:** aortic aneurysm, cardiology, cardiothoracic surgery, decision making, sinus of valsalva aneurysm

## Abstract

A sinus of Valsalva aneurysm (SOVA) is an aneurysm of the aortic root between the aortic valve annulus and the sinotubular junction. Unruptured SOVAs are often asymptomatic and discovered incidentally during cardiac imaging. Rupture of a SOVA can lead to acute or insidious hemodynamic compromise. When SOVAs are diagnosed, they are accompanied by the decision to recommend surgical intervention versus conservative medical therapy. We are reporting a challenging case of a young female patient presenting with an incidental finding of an aneurysm of the sinus of Valsalva.

A previously healthy 21-year-old woman with no prior medical history was referred for an evaluation of recurrent syncopal episodes. She was otherwise healthy without any pertinent medical history. An incidental finding of an aneurysm of the sinus of Valsalva involving the right coronary cusp was noted, measuring 11 mm (width) x 10 mm (depth) on the echocardiogram. The patient presented a clinical decision dilemma regarding the best strategy for treatment and follow-up. She was referred to cardiothoracic surgery for further evaluation. Her young age, unusual presentation, and remaining asymptomatic contributed to the decision to continue with a conservative approach.

She has continued to be evaluated in the outpatient setting by serial echocardiograms. These studies have remained stable without progression of the aneurysm. After two years of close follow-up, the patient has demonstrated continued stability of the aneurysm. In light of the guidelines regarding aortic disease, it remains of the utmost importance to keep patients and their presenting symptoms at the center of decision-making. Conservative management with close outpatient follow-up was successful in keeping our young asymptomatic patient and her aneurysm stable. However, there remains a role for surgical intervention, when appropriate, in other clinical scenarios.

## Introduction

A sinus of Valsalva aneurysm (SOVA) is an aneurysm of the aortic root between the aortic valve annulus and the sinotubular junction [1]. SOVAs are rare, occurring in 0.09% of the general population, based on autopsy series. They are involved in up to 3.5% of all congenital heart defects. Typically, the right coronary sinus is affected, followed by the noncoronary sinus, and lastly, the left coronary sinus. It also affects men four times more frequently than women [1].

Approximately 20% of thoracic aortic aneurysms are genetic and present at younger ages, while a large portion of aortic root aneurysms may be sporadic and idiopathic [2]. Congenital SOVAs typically result from a structural weakness at the junction of the aortic media and the annulus fibrosus, whereas acquired forms may arise secondary to infections, trauma, or connective tissue disorders [3,4]. This is in contrast to descending thoracic aortic disease, which tends to be degenerative and present at older ages. Due to these findings, individuals with aortic root or ascending aortic aneurysms should have first-degree relatives screened [5].

Unruptured SOVAs are often asymptomatic and discovered incidentally during cardiac imaging, but may present with symptoms due to mass effect on adjacent structures, including the coronary arteries and cardiac valves [4-7]. Rupture of an SOVA can lead to acute or insidious hemodynamic compromise, manifesting as aorto-cardiac shunt, congestive heart failure, or, in severe cases, cardiac arrest [3,6,8]. The risk factors and predictors for rupture remain incompletely understood, but the high morbidity and mortality associated with rupture underscore the need for early recognition and management [3,5].

When SOVAs are diagnosed, they are accompanied by the decision to recommend surgical intervention versus conservative medical therapy. The rarity of SOVAs, combined with the absence of established management guidelines, necessitates individualized clinical decision-making based on limited evidence from case reports and small series rather than controlled trials. We are reporting a challenging case of a young female patient presenting with an incidental finding of an aneurysm of the sinus of Valsalva.

## Case presentation

A previously healthy 21-year-old woman with no prior medical history was referred for an evaluation of recurrent syncopal episodes. The patient reported feeling warm and lightheaded while watching medical education videos as part of her training as a dental hygienist student. The patient was told she had a murmur as a child, but her family could not remember details with regard to what workup was performed. She was otherwise healthy without any pertinent medical history. She denied syncopal episodes while performing other activities of daily living or exercise. She denied having chest pain, dyspnea on exertion, palpitations, lower extremity edema, trauma following syncope or family history of sudden cardiac arrest/death. The patient did not participate in competitive sports.

**Figure 1:**
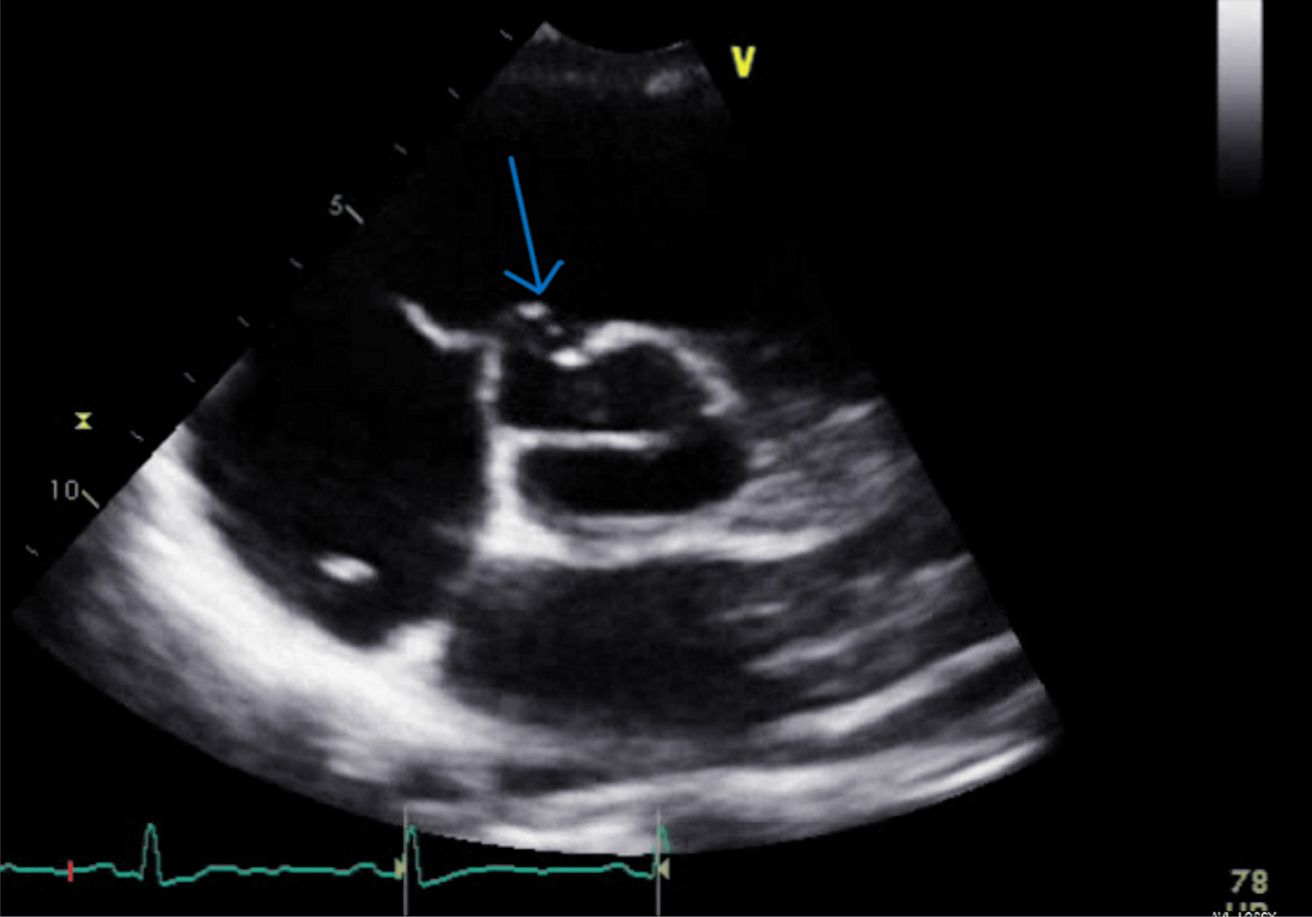
Short axis view at the level of the aortic valve from transthoracic echocardiography. The blue arrow indicates an aneurysm of the sinus of Valsalva involving the right coronary cusp, measuring 11 mm (width) x 10 mm (depth). The image was taken at end systole/early diastole in the cardiac cycle.

**Figure 2:**
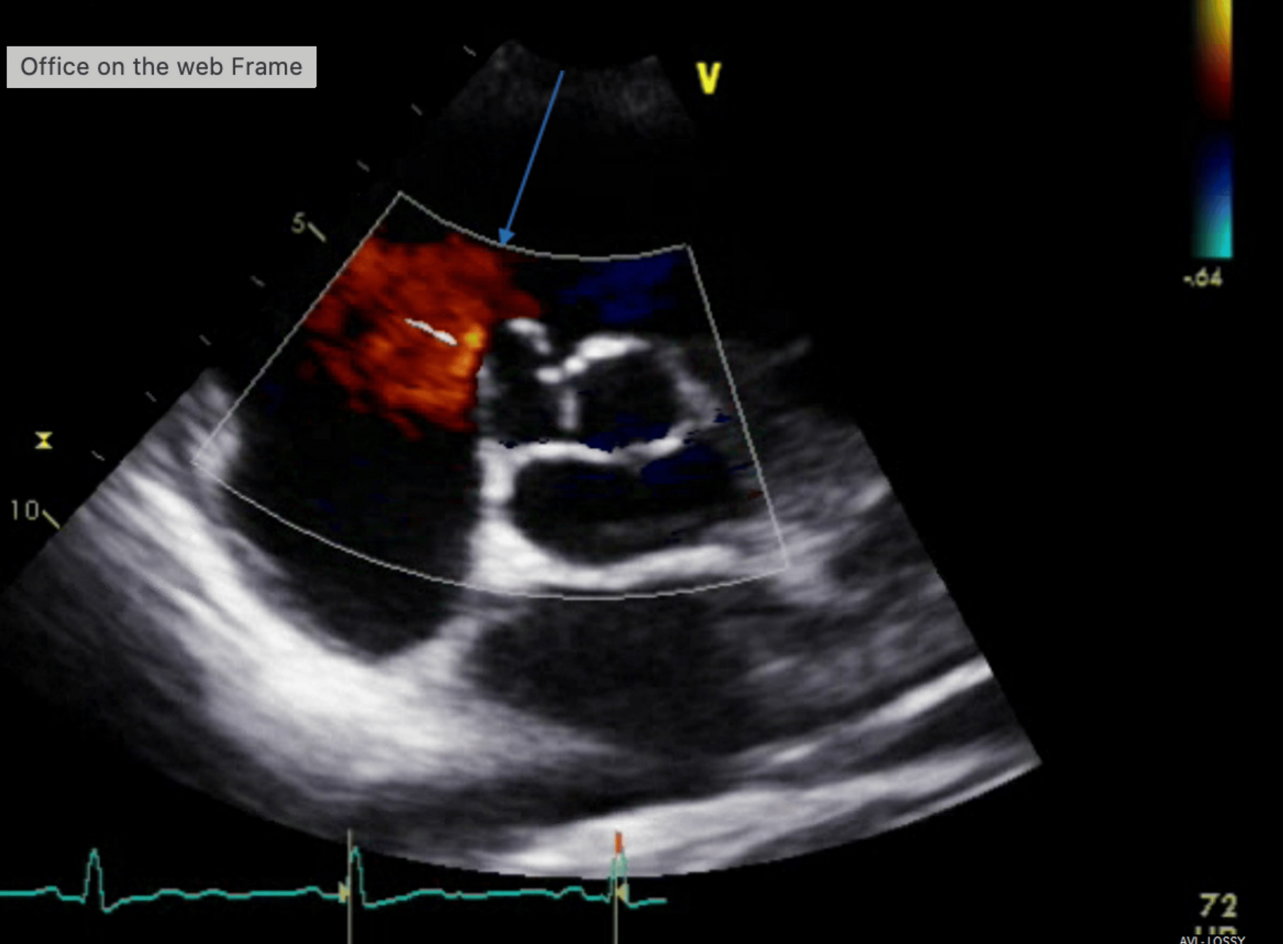
Short axis view at the level of the aortic valve from transthoracic echocardiography There is color Doppler flow overlying, displayed in red. The color flow is not crossing over from the right atrium/right ventricle into the sinus of Valsalva aneurysm, demonstrating a non-ruptured aneurysm. The blue arrow indicates the sinus of Valsalva aneurysm involving the right coronary cusp, measuring 11 mm (width) x 10 mm (depth). The image was taken during end diastole of the cardiac cycle.

A 12-lead ECG revealed normal sinus rhythm with normal intervals. An echocardiogram demonstrated normal septal thickness and normal left and right ventricular systolic function without any overt valvular regurgitation or stenotic lesions. However, an incidental finding of an aneurysm of the sinus of Valsalva involving the right coronary cusp was noted, measuring 11 mm (width) x 10 mm (depth) (Figures 1, 2).

This patient presented a complex clinical decision-making dilemma regarding optimal therapeutic strategy and surveillance protocol. Following cardiothoracic surgery consultation, a comprehensive evaluation was undertaken to elucidate the etiology of her syncope. This workup included orthostatic vital sign assessment, which demonstrated no evidence of orthostatic hypotension. Also, a 30-day cardiac event monitor was negative for arrhythmic abnormalities. Given the constellation of her young age, atypical presentation with syncope, absence of symptoms directly attributable to the aneurysm, and lack of established surgical indications, a conservative management strategy with close surveillance was adopted.

Serial transthoracic echocardiography has been performed at regular intervals. These serial echocardiography studies have consistently demonstrated stability of the aneurysm without evidence of progression, development of aortic regurgitation, or other complications. After 24 months of rigorous outpatient follow-up, the patient has remained clinically asymptomatic with continued anatomic stability of the sinus of Valsalva aneurysm, supporting the appropriateness of the conservative approach in this carefully selected case.

## Discussion

The finding of an aneurysm of the sinus of Valsalva in this otherwise healthy young woman was largely incidental and did not explain her clinical presentation of recurrent syncopal episodes. These syncopal episodes were deemed more consistent with neurocardiogenic syncope in etiology. Due to this, the decision regarding referral for surgical management versus conservative medical therapy became challenging.

​​​When considering management, size, location, and speed of enlargement are important factors [1]. Symptoms are often related to the mass effect of the aneurysm or sequelae of rupture [3]. One retrospective study found the rate of rupture to be 34% [9]. Rupture of aneurysms involving the right and non-coronary cusps will usually result in a shunt between the aorta and right ventricular outflow tract or right atrium, while rupture of an aneurysm involving the left coronary cusp will result in a communication between the aorta and left ventricular outflow tract or left atrium [10,11]. Additionally, large aneurysms can also be a nidus for thrombus formation [3].

The rate of rupture for a SOVA is reported in the medical literature to be approximately 34% to 49% among patients presenting with this condition [12]. These data reflect the natural history in referred or surgically managed cohorts and may overestimate the rupture rate compared to incidentally discovered, asymptomatic aneurysms [13]. However, there may be limited use of extrapolating these data as the SOVA in our patient was noted to be small and incidental. No specific guidelines are currently available for the management of isolated aneurysms involving the sinus of Valsalva. However, it may be reasonable to follow the 2022 ACC/AHA (American College of Cardiology and American Heart Association) guidelines regarding aortic disease [12].

Imaging

Evaluation and subsequent monitoring of ascending aortic aneurysms can be done with various imaging modalities, including echocardiography, computed tomography (CT), or magnetic resonance imaging (MRI). The guidelines emphasize the importance of reproducible and accurate measurements of the aorta for characterizing disease and guiding treatment. Transthoracic echocardiography (TTE) has been historically used for the evaluation of the aortic root, and has led to the determination of the normal limits adjusted for age, sex, and body size [13]. TTE has a good correlation with measurements obtained on CT and MRI [14]. The benefit of CT and MRI is lower interobserver and intra-observer variability in determining aortic root margins [15]. In patients with dilated thoracic aortas, there is a class 1 recommendation to use transthoracic echocardiography to assess aortic valve anatomy and function, and thoracic aortic diameter. The recommendation for CT or MRI is class 2a, and there is a 2a recommendation for follow-up imaging in 6 to 12 months to determine the rate of growth, and if stable, surveying every 6 to 24 months is recommended [12].

Medical therapy

The primary goal of medical therapy in aortic disease is to reduce growth rate, risk of aortic disease-related mortality, and the need for surgical or endovascular repair. The secondary goal of medical management is the prevention of non-aortic cardiovascular disease given shared risk factors [16]. Many of the medical management pathways for aortic root disease are driven by studies performed on abdominal aortic aneurysms or Marfan syndrome. These pathways converge with the common goal of reducing aortic vascular remodeling. This is accomplished through the reduction of aortic wall stress, which may mitigate proteolysis pathways that lead to medial degeneration, as well as possible pleiotropic effects of statins [7].

Currently, there are no clinical trials regarding the management of hypertension in patients with thoracic aortic disease, but the 2022 AHA/ACC guidelines outline a class 1 indication for all patients with cardiovascular disease to have the blood pressure goal less than 130/80 mmHg [16]. However, systolic blood pressure less than 120 mmHg may be beneficial [12].

The benefits of beta blockers in aortic root aneurysms are largely derived from studies on patients with Marfan syndrome. Beta blockers may reduce the rate of aortic root aneurysm growth and reduce aortic root complications [17,18]. Similarly, in a study, angiotensin-converting enzyme (ACE) inhibitors were found to have no difference in outcomes compared to beta blockers in Marfan patients and were found to be superior to placebo, and a combination of ACE inhibitors and beta blockers had better outcomes than beta blockers alone [19]. For the use of beta blockers and angiotensin receptor blockers, the recent 2022 guidelines offer a class 2a recommendation for thoracic aortic aneurysm in the absence of contraindications, regardless of the underlying cause [12].

Statins have been hypothesized to have a pleiotropic effect in reducing vascular remodeling associated with thoracic aortic aneurysm, thereby reducing the rate of growth regardless of the underlying cause, even in the absence of atherosclerosis. The use of aspirin is a class 2a recommendation, with atherosclerotic thoracic aortic aneurysm with aortic atheroma or penetrating aortic ulcer [12].

All patients with cardiovascular disease, including aortic root and sinus of Valsalva aneurysms, should be counseled on smoking cessation and offered therapeutic modalities that may help facilitate this. Lifestyle-modifying discussions with patients should include conversations on physical activity. As class 1 recommendations, patients with significant aortic disease should avoid intense isometric exercises that require the Valsalva maneuver, burst exertion, and collision sports [12].

Surgical therapy

Surgery is indicated as a class 1 recommendation for aneurysms of the aortic root and ascending aorta in symptomatic patients. Additionally, repair for asymptomatic patients with the aortic root or ascending aorta diameter ≥5.5 cm is a class 1 recommendation. For patients with diameters less than 5.5 cm whose growth rate is confirmed by imaging as ≥0.3 cm/year for two consecutive years, or ≥5.5 cm in one year, there is a class 1 recommendation for surgical repair. Asymptomatic patients with a diameter ≥5.0 cm may undergo repair performed by experienced surgeons in a multidisciplinary aortic team, with a class 2a recommendation. Lastly, asymptomatic patients with an aortic size index, or ASI (aortic diameter/body surface area (BSA)) ≥3.08 cm/m^2^, or aortic height index, or AHI (aortic diameter/height) >3.21 cm/m may undergo surgical repair performed by a multidisciplinary aortic team under a class 2b recommendation [12].

The evaluation of asymptomatic patients should include candidacy for aortic repair. The purpose of surgical or endovascular repair is to reduce the risk of adverse aortic events, including dissection or rupture. These evaluations weigh the risk of future events against interventional risks. For open surgical options, the aneurysmal segment is replaced with a prosthetic graft. Endovascular repair includes stenting across the aneurysmal segment. Currently, there are FDA-approved stents for the descending thoracic aorta, with stent grafts for the ascending aorta, aortic arch, and thoracoabdominal aorta in clinical trials in the United States [20].

## Conclusions

In light of the guidelines regarding aortic disease, it remains of the utmost importance to keep patients and their presenting symptoms at the center of decision-making. SOVAs are generally rare, asymptomatic, and found incidentally on echocardiography. Conservative management with close outpatient follow-up was successful in keeping our young asymptomatic patient and her aneurysm stable. However, there remains a role for surgical intervention, when appropriate, in other clinical scenarios.
